# Supplementary material for: Towards Ecological Management and Sustainable Urban Planning in Seoul, South Korea: Mapping Wild Pollinator Habitat Preferences and Corridors Using Citizen Science Data
Source: Animals (Basel). 2022 Jun 6;12(11):1469. doi: 10.3390/ani12111469 (PMC9179275; doi:10.3390/ani12111469)
Supplement: Supplementary file 1 [file animals-12-01469-s001.zip › animals-1591099-supplementary.pdf]

Supplementary material.

Table S1. Dispersion distance attributed to the six families of wild pollinators according to the literature.

Family	Mean dispersion distance	References
Apidae (*Apis mellifera* and *Apis cerana* excluded)	1000 m	Zurbuchen et al.; 2010; Knight et al., 2005; Jauker et al., 2009; Walter et al., 2000
Halictidae	200 m	Zurbuchen et al., 2010; Penone et al., 2012; Gathmann and Tscharntke, 2002;
Megachilidae	500 m	Zurbuchen et al., 2010; Vicens and Bosh, 2000;
Syrphidae	1000 m	Jauker et al., 2009
Pieridae	3000 m	Bergerot et al., 2013; Cowley, 2001
Lycaenidae	500 m	Bergerot et al., 2010; Cowley, 2001
